# Asthma-related deaths in Brazil: data from an ecological study

**DOI:** 10.36416/1806-3756/e20240296

**Published:** 2024-12-30

**Authors:** Amanda da Rocha Oliveira Cardoso, Anna Carolina Galvão Ferreira, Marcelo Fouad Rabahi

**Affiliations:** 1. Departamento de Pneumologia, Hospital das Clínicas, Programa de Pós Graduação em Ciências da Saúde, Faculdade de Medicina, Universidade Federal de Goiás, Goiânia (GO), Brasil.

**Keywords:** asthma, mortality, hospitalization, epidemiology

## Abstract

**Objective:**

The aim of this study was to present epidemiological data on hospitalizations and deaths related to asthma in Brazil over the past 11 years.

**Methods:**

An ecological study was conducted on asthma-related hospitalizations and mortality in Brazil from 2013 to 2023, using data extracted from the Department of Informatics of the Brazilian Unified Health System and the Mortality Information System.

**Results:**

Asthma-related deaths showed an increasing trend during the analyzed period. A surge in deaths was observed in 2022 compared to 2014 (difference between means = 56.08 ± 19.7; 95% CI = 15.2-96.9). The mean number of deaths was higher among females, with their rate remaining stable, while the rate for males increased. Individuals aged >60 years accounted for approximately 65% of all asthma-related deaths from 2013 to 2023, with a strong direct correlation observed between age and the number of deaths, regardless of sex. During the same period, the total number of asthma-related hospitalizations in Brazil showed a declining trend, decreasing from 134,322 in 2013 to 87,707 in 2023.

**Conclusion:**

Over the past 11 years, asthma-related deaths have increased in Brazil, with the majority occurring among females. Older individuals accounted for most asthma-related deaths, and a positive correlation was observed between age and the number of deaths.

## INTRODUCTION

Asthma affects approximately 339 million individuals worldwide.^1^ In Brazil, the prevalence of asthma symptoms is estimated at 23% among adolescents and adults.^2^ As with other chronic non-communicable diseases, most asthma patients should be managed in primary care, typically requiring low doses of inhaled corticosteroids for disease control.^3^ Despite the universal and free availability of inhaled corticosteroids for the treatment of asthma in Brazil, the number of hospitalizations and deaths remains high.^4^ In 2013 alone, 2,047 individuals died of asthma in the country, equating to five asthma-related deaths per day.^5^ These deaths are preventable, highlighting the need for improved management of this condition. Reliable and valid data are critical for objective health situation analysis, decision-making, and health action planning. Measuring the health status of a population is a fundamental public health activity, relying on the systematic recording of mortality and survival data.^6^ In this context, the aim of our study was to present epidemiological data on asthma-related deaths and hospitalizations in Brazil over the past 11 years.

## METHODS

This ecological study analyzed asthma-related mortality and hospitalizations in Brazil between 2013 and 2023. Data were extracted from the official databases of the Mortality Information System (SIM)^7^ and the Department of Informatics of the Unified Health System (DATASUS).^8^ Population and geographic indicators were obtained from the Brazilian Institute of Geography and Statistics (IBGE) censuses^9^ of 2010 and 2022, as well as from available population projections for historical series. Data collection took place in June 2024. 

Data on asthma-related deaths were extracted exclusively from SIM,^7^ which records deaths that occurred in both public and supplementary healthcare systems, as well as those outside hospital settings. The selected parameters included: “reference years” (2013 to 2023), “place of record” (deaths by place of residence), “indicator” (International Classification of Diseases [ICD]-10 codes J45 [asthma] and J46 [status asthmaticus]), “age group” (<1, 1-4, 5-9, 10-14, 15-19, 20-29, 30-39, 40-49, 50-59, 60-69, 70-79, and >80 years), and sex (male or female). These parameters were used to generate tables with annual asthma-related death numbers on the Integrated Health Surveillance Platform of the Ministry of Health. The data for 2023 are preliminary. 

Absolute numbers of hospitalizations, mean/total hospitalization costs, and mean length of stay per year were derived from the “Epidemiology and Morbidity” tables under the “Hospital Morbidity of SUS” section, “General by Place of Hospitalization,” for the geographic scope “Brazil by Region and State - from 2008.” These tables were generated by the Integrated Health Surveillance Platform of the Ministry of Health, using DATASUS^8^ as the data source. The options selected included “year/month of service,” “age group,” “hospitalizations,” “total cost,” “mean hospitalization cost,” “mean length of stay,” and “asthma” in the morbidity list on the platform. It is important to note that DATASUS^8^ data refer exclusively to patients hospitalized within SUS. The second half of the 2023 data, according to DATASUS, was subject to updates. Regarding hospitalization costs, the monetary value of each hospitalization is provided by DATASUS and is based on billed Hospital Admission Authorizations (HAAs) that contain the studied ICD codes. Hospitalization cost values in Brazilian real (R$) were converted to USD using the exchange rate of R$ 1.00 = USD 5.66, as of July 3, 2024. 

The asthma mortality rate was calculated using the formula: (absolute number of asthma-related deaths × 100,000)/population per year.^10^ Population estimates for each year were calculated based on an annual population growth rate of 0.52% for the periods 2013-2021 and 2023. The populations reported in the IBGE census^9^ ^)^ were used for 2010 and 2022. The same population growth rate was applied to both sexes. 

Normality was assessed using the Shapiro-Wilk test. Analysis of variance (ANOVA) was performed to test for significant variations in total deaths and deaths according to sex, with the Bonferroni test used to identify where differences between means occurred. Non-parametric tests were used to analyze hospitalization costs. The Kruskal-Wallis test was applied to assess differences in total and mean hospitalization costs, as well as hospitalization rates over the study period, according to age group. Linear regression analysis was conducted to determine whether the total cost value (number or mean cost of hospitalizations) explained the hospitalization costs; the R² and the 95% confidence interval were used to determine the accuracy of the results. Spearman’s correlation coefficient was calculated to analyze the relationship between age and mortality. All analyses were performed using Jamovi^®^ version 2.2, Orange^®^ version 3.37, and Stata^®^ version 16.0 software, with the significance level set at 5%.

### Ethical Aspects

Ethical review was not required for this study, as it involved a retrospective analysis of anonymized data in the public domain, aligning with CNS Resolution 466/12. Also, this study did not receive any funding.

## RESULTS

### Asthma Mortality

**Figure 1:**
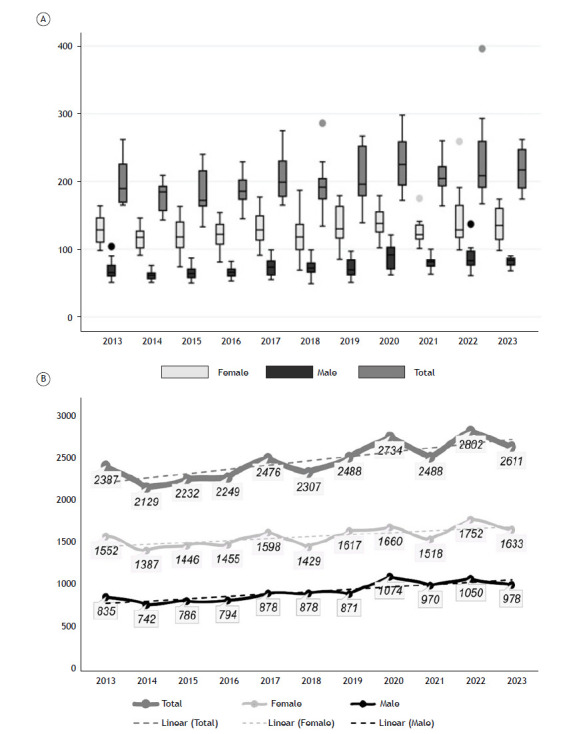
(a) Box Plot: Mortality trends considering the monthly median per year between 2013 and 2023, with variations by sex (male vs. female). Mean number of deaths among females were higher than those among males (p < 0.005). (b) Annual quantitative mortality trends by sex (male vs. female) between 2013 and 2023.

Between 2013 and 2023, asthma-related deaths showed an overall increase, as illustrated in Figures 1a and 1b. Notably, there was a rise in deaths in 2020, totaling 2,734, followed by a decline in 2021 (2,488 deaths), another increase in 2022 (2,802 deaths), and a slight decrease in 2023 (2,611 deaths). Despite these fluctuations, the number of deaths remained higher compared to 2013. 

Female mortality was consistent throughout the analyzed period (2013-2023; p = 0.16). In contrast, significant variation was observed in male mortality, as determined by ANOVA with Bonferroni correction (Table 1). Considering both sexes, a notable increase in deaths was identified when comparing 2014 to 2022, with a difference between means (DM) of 56.08 ± 19.7 (95% confidence interval [CI] = 15.2-96.9; p = 0.02).

**Table 1:** Significant differences in mean deaths considering annual and sex comparisons.

Statistically significant differences	DM ± SD	95% CI	p-value
Male 2013 x 2020	19.91 ± 7.45	4.46 - 35.4	0.02
Male 2014 x 2020	27.66 ± 6.34	14.5 - 40.8	0.0002
Male 2015 x 2020	24 ± 6.67	10.2 - 37.8	0.002
Male 2016 x 2020	23.33 ± 6.36	10.1 - 36.5	0.003
Male 2014 x 2021	19 ± 3.82	11.1 - 26.9	0.05
Male 2014 x 2022	25.66 ± 6.25	12.7 - 38.6	0.001
Male 2015 x 2022	22 ± 6.59	8.34 - 35.7	0.008
Male 2016 x 2022	21.33 ± 6.28	8.31 - 34.4	0.01
Male and Female 2014 x 2022	56.08 ± 19.7	15.2 - 96.9	0.02

DM= Difference between means; SD= Standard deviation; CI= Confidence interval. Results with statistically significant differences.

The increase in deaths among males between 2020 and 2023 explains the overall rise in asthma-related deaths. Despite this increase, the mean number of deaths among females was higher than that observed among males in all years (p < 0.005) (Figures 1a and 1b). 

The overall asthma mortality rates per 100,000 inhabitants remained stable between 2013 and 2023. However, an upward trend was observed in male mortality rates over the same period (Table 2). 

**Table 2:** Annual mortality rates per 100,000 inhabitants in Brazil.

Year	Total	Male	Female
2013	1.23	0.88	1.56
2014	1.09	0.77	1.39
2015	1.14	0.81	1.44
2016	1.14	0.82	1.44
2017	1.25	0.90	1.58
2018	1.16	0.90	1.40
2019	1.24	0.88	1.58
2020	1.36	1.09	1.61
2021	1.23	0.98	1.47
2022	1.38	1.06	1.67
2023	1.19	0.98	1.55

Considering the distribution of deaths by sex, the majority occurred in females (63%). However, among children and adolescents (≤19 years), mortality was higher in males than in females. Between the ages of 15 and 29 years, the number of deaths was nearly equal between sexes. From age 30 onward, deaths were more frequent among women. 

**Figure 2:**
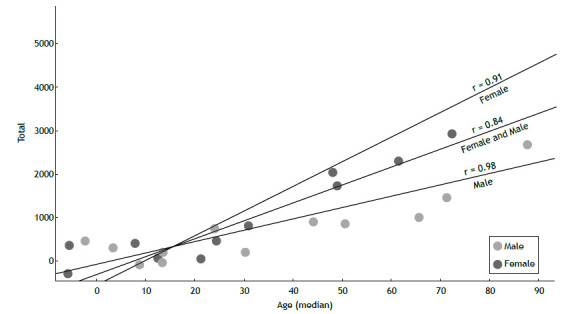
Spearman’s correlation: relationship between median age and the number of deaths by sex (dark grey: female; light gray: male; black: both sexes).

In the age-stratified analysis, we observed a progressive increase in asthma-related mortality with age, with older age groups having the highest mortality rates (Figure 2). The highest proportion of deaths occurred in the >70 (31.65%) and >80 years (31.65%) age groups. Individuals aged over 60 accounted for approximately 65% of all asthma-related deaths during the study period. Mortality among children aged <1 year was relatively low compared to other age groups, representing only 0.60% of the total deaths. Although it represented a small proportion of the total deaths, the 20-39 years age group accounted for approximately 6-9%. Furthermore, a strong positive correlation was observed between age and the number of deaths, regardless of sex (Figure 2).

#### Asthma-related hospitalizations in SUS

**Figure 3:**
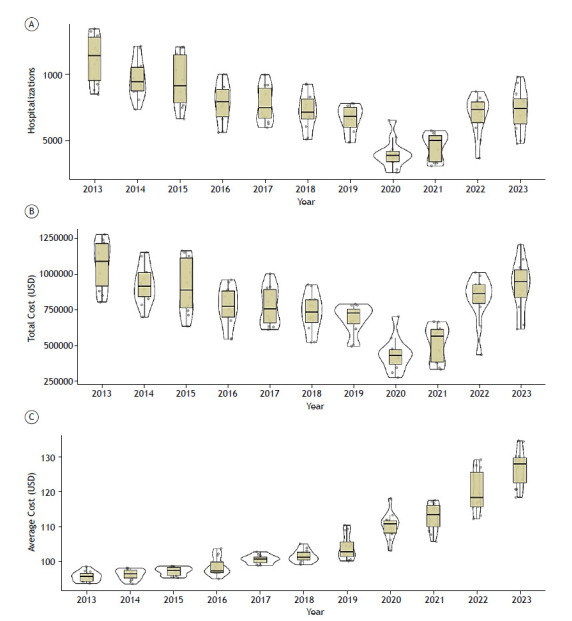
Box Plot graph, with means and medians, and violinplot to determine where the most accumulated data is. (a) Total number of hospitalizations due to asthma in SUS between 2013 and 2023; (b) Total cost of hospitalizations due to asthma in SUS between 2013 and 2023; (c) Mean hospitalization costs due to asthma in SUS between 2013 and 2023. Note the significant drop in asthma-related hospitalizations in 2020 (p < 0.05).

Between 2013 and 2023, the total number of asthma-related hospitalizations in SUS in Brazil showed a declining trend, decreasing from 134,322 hospitalizations in 2013 to 87,707 in 2023 (Figure 3a). A significant decline in asthma-related hospitalizations was observed in 2020 compared to other years (p-value < 0.05), followed by an increase in subsequent years. By 2023, the numbers returned to the pattern seen during the pre-pandemic period. The mean length of hospitalization in SUS was 3 days throughout the study period.

According to DATASUS, the mean cost of asthma-related hospitalizations during the study period gradually increased, rising from USD 95.25 in 2013 to USD 126.65 in 2023 (Figure 3c). 

**Figure 4:**
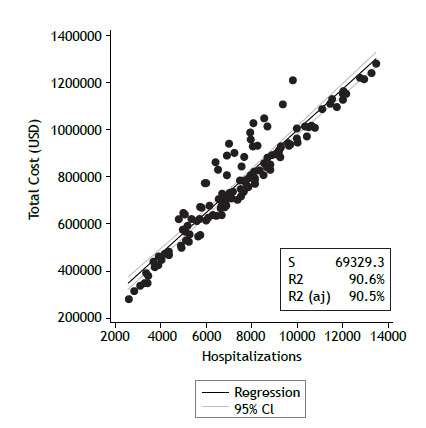
Linear regression. Association between the number of hospitalizations and the total cost. Monthly dispersion. R² = 0.90; p < 0.001.

The total cost of hospitalizations in SUS (Figure 3b) showed a strong positive correlation (r = 0.95; p < 0.001) with the number of hospitalizations. In the linear regression analysis, the number of hospitalizations explained 90% of the total cost (R² = 0.9; p < 0.001) (Figure 4).

#### Asthma-related hospitalizations by age group

Regarding asthma-related hospitalizations across the different age groups, the increase observed in 2021 and 2022 occurred mainly in individuals younger than 14 years of age. Notably, hospitalizations in the 5-9 years age group rose by 85% in 2022 compared to the previous year. In contrast, in the 20-29 and 30-39 years age groups, hospitalizations decreased slightly during the same period. Among individuals aged >60 years, there was a noticeable increase in hospitalizations in 2021 and 2022 compared to 2020, with the largest rise observed in the 70-79 years age group. This age group accounted for 32% of all hospitalizations in the last two evaluated years. The number of asthma-related hospitalizations remained stable in 2023 compared to 2022. 

## DISCUSSION

We observed variation in the absolute number of asthma-related deaths in Brazil over the past 11 years, with a notable upward trend. Comparing 2014 and 2022, the mean number of asthma-related deaths per year was 2,445, corresponding to approximately 6.76 deaths per day. A similar study^5^ conducted between 2008 and 2013 reported 2,047 deaths in 2013, equating to approximately five deaths per day, along with a 10% reduction in total deaths during the analyzed period. In contrast, our study showed an increase in the number of deaths from 2019 to 2022, with the number in 2023 being similar to that in 2013. These findings do not support a trend of reduction in the absolute number of asthma-related deaths in Brazil over the past 11 years. Notably, the mentioned study^5^ analyzed only data associated with ICD-10 J45. In contrast, our study also included ICD-10 J46 (status asthmaticus), which accounted for 6-14% of the total deaths over the past 11 years.

Recently, an article was published reporting mortality data in Brazil between 2008 and 2021, identifying 8,497 asthma-related deaths during that period and a decrease in the number of deaths over time.^11^ However, that study relied solely on data from DATASUS,^8^ which reflects deaths based on HAA forms coded as asthma within SUS. In contrast, the SIM system^7^ (the source of our data) uses Death Certificates (DCs) as the source document, thereby providing official and more comprehensive asthma-related death data. DATASUS shows HAA data for asthma-related hospitalizations concluded with the outcome “discharge by death,” making its mortality data incomplete. SIM, on the other hand, reports the cause of death as listed on the DC, regardless of the HAA coding, place of death (hospital or home), or whether it occurred in the public or supplemental healthcare system. Thus, the SIM system offers a more reliable representation of asthma-related mortality in Brazil.

Mortality rates in the country are a critical public health concern. Despite government efforts to provide free medications, a significant number of asthma-related deaths have been observed among the Brazilian population. This study did not aim to analyze the causes of increased mortality; therefore, we can only speculate on the contributing factors. Since 2009, Brazil has implemented a policy for free access to medications through the “Popular Pharmacy” program, which includes the provision of inhaled corticosteroids (beclomethasone) and short-acting bronchodilators (salbutamol).^12^ Additionally, since 2002, the Ministry of Health has supplied free, high-cost medications-such as budesonide, the combination of budesonide-formoterol, and biologics like mepolizumab and omalizumab (for severe cases)-to support asthma treatment.^13^

Inhaled corticosteroids are essential for the treatment of asthma^3^ and for reducing asthma-related mortality.^14^ However, asthma management involves several challenges, including low treatment adherence, difficulties with inhalation techniques, limited health education, and poor health literacy among the population. Moreover, asthma management has evolved considerably in recent decades, and implementing complex guidelines for primary and secondary care professionals remains challenging, requiring the availability of practical management guides^15^ and the establishment of ongoing medical education programs, particularly for primary care professionals, who are responsible for managing most patients with asthma.

In the present study, we observed that the highest number of deaths occurred between 2020 and 2022, coinciding with the coronavirus disease (COVID-19) pandemic.^16^ A systematic review and meta-analysis^17^ involving 131 studies and over 410,000 patients found no association between asthma and increased COVID-19 severity or worse outcomes in infected individuals. However, the use of systemic corticosteroids for asthma exacerbation treatment-whether recent (within the past 120 days) or frequent (two courses of 40 mg/day for at least 3 days per year)-was identified as an independent risk factor for increased severity of COVID-19 and higher mortality.^18^

We can speculate that the increase in asthma-related deaths during the pandemic period occurred among severely ill patients who frequently used systemic corticosteroids or among mildly ill patients who faced disruptions in routine medical care due to social isolation measures, lost access to asthma control medications, or discontinued regular use of systemic corticosteroids.

Most deaths occurred among females. Throughout the observation period, the mean number of deaths among females was higher than that among males. Until 2010, the asthma mortality rate per 100,000 inhabitants in Brazil, calculated based on data from DATASUS, was higher in females than in males (females: 0.241 vs. males: 0.193 per 100,000).^19^ In 2022, according to SIM data, we observed a higher mortality rate compared to 12 years earlier (females: 1.67 vs. males: 1.06). However, the earlier data^19^ were calculated exclusively using DATASUS, while our findings were based on SIM data, using the distinct methodological characteristics described previously.

The prevalence of asthma varies with age, being more pronounced in males during childhood and puberty, but more prevalent in females during adulthood.^20^ Factors such as sex hormones, genetic and epigenetic variations, social and environmental influences, and differences in therapeutic responses contribute to the observed disparities between sexes in terms of asthma incidence, prevalence, and severity. Severe asthma is more common in females than in males, and females tend to exhibit poorer asthma control and more frequent exacerbations compared to males.^21^

However, the increase in male mortality observed in recent years is noteworthy. This trend may be attributed to lower healthcare-seeking behaviors among males, as well as potential shortcomings in asthma management within the healthcare system.

Older individuals account for 66% of asthma-related deaths in Brazil, with a positive correlation between age and the number of deaths, regardless of sex. Compared to adult asthma patients, older patients experience more frequent and severe exacerbations, worse lung function, and a higher prevalence of comorbidities,^22^ all of which may contribute to the elevated mortality in this age group. Older asthma patients are also more likely to be underdiagnosed and, as a result, inadequately treated.^23^ Notably, a decade ago, older patients had higher rates of emergency department utilization, greater mortality, more hospitalizations, and longer hospital stays compared to adult asthma patients.^24^

The number of asthma-related hospitalizations in Brazil through SUS^8^ has decreased over the past 11 years, dropping from over 130,000 in 2013 to approximately 87,000 in 2023. During this period, the number of inpatient pulmonology beds for adults declined from 1,822 to 1,622,^8^ while Brazil’s population grew by nearly 7.5%.^9^ This suggests that the reduction in hospitalizations may be attributed to a decrease in available resources rather than an improvement in asthma care.

Approximately 66% of the hospitalizations occurred in the pediatric age group (under 14 years). However, the number of pediatric beds also decreased by over 10,000 during this period, from 44,060 in January 2013 to 32,980 in January 2023.^8^ This reduction in available beds may explain the decline in hospitalizations, rather than a true improvement in asthma-related outcomes.

In 2020, during the COVID-19 pandemic, a sharp decline in asthma-related hospitalizations was observed in Brazil. At that time, care patterns were restructured to prioritize the treatment of severe COVID-19 cases, leading to a greater allocation of hospital beds for these patients. Despite the reduction in the absolute number of asthma-related hospitalizations and the stability of the mean length of stay (3 days), the mean cost per asthma-related hospitalization increased progressively. However, the total cost of hospitalizations was not primarily driven by this rise in the mean cost but was instead influenced by the overall reduction in the number of hospitalizations.

In Brazil, approximately 75% of the population relies on SUS, while the remainder uses the Supplemental Healthcare System.^8^ Consequently, the reduction in hospital bed availability significantly impacts a large portion of the population. An inverse relationship has been observed between the rise in asthma-related deaths and the decline in available hospital beds. According to SIM,^7^ around 60% of asthma-related deaths from 2013 to 2023 occurred in hospitals (SUS and supplemental), 10% in other healthcare settings, and 27% at home. These findings suggest that with fewer hospital beds, patients with more severe conditions may be prioritized for admission. The high proportion of deaths occurring outside hospitals is particularly concerning, as it indicates gaps in asthma care, both in maintenance and acute treatment. This highlights the urgent need for public measures to train healthcare professionals in asthma management, especially in emergency units nationwide.

This study had some limitations. First, as an ecological study, it was not possible to establish direct relationships between individual-level variables. Instead, we analyzed the variables collectively and conducted a literature review to explore potential explanations. Another limitation was the reliance on secondary data sources, which raises the possibility of diagnostic errors, particularly in distinguishing asthma from chronic obstructive pulmonary disease (COPD)-a common challenge in older individuals.^23^ It is important to note, however, that asthma is frequently underdiagnosed in this population. Therefore, if diagnostic errors occurred, mortality and hospitalization rates for individuals over 60 years of age are likely even higher.

Despite these limitations, our data provide valuable insights into asthma mortality rates in Brazil. We believe these findings can play a crucial role in informing the planning and implementation of public health policies for asthma management.

In conclusion, asthma-related deaths in Brazil have increased over the past 11 years, particularly between 2014 and 2022. The majority of deaths occurred in females. Older individuals also accounted for most asthma-related deaths, with a positive correlation observed between age and the number of deaths, regardless of sex. Meanwhile, the number of asthma-related hospitalizations in Brazil has decreased over the same period.
